# Prognostic role of margin status in open and CO_2_ laser cordectomy for T1a–T1b glottic cancer^☆^

**DOI:** 10.1016/j.bjorl.2016.11.006

**Published:** 2016-12-24

**Authors:** Vincenzo Landolfo, Carmine Fernando Gervasio, Giuseppe Riva, Massimiliano Garzaro, Rita Audisio, Giancarlo Pecorari, Roberto Albera

**Affiliations:** a University of Turin, Surgical Sciences Department, 1st ENT Division, Turin, Italy; b University of Turin, Surgical Sciences Department, 2nd ENT Division, Turin, Italy

**Keywords:** Laryngeal neoplasms, Early glottic cancer, Margin status, Overall survival, Disease free survival, Neoplasias laríngeas, Câncer glótico inicial, Estado de margem, Sobrevida global, Sobrevida livre de doença

## Abstract

**Introduction:**

Cordectomy by laringofissure and transoral laser surgery has been proposed for the treatment of early glottic cancer.

**Objectives:**

The aim of this retrospective study was to evaluate the prognostic value of margin status in 162 consecutive cases of early glottic carcinoma (Tis–T1) treated with CO_2_ laser endoscopic surgery (Group A) or laryngofissure cordectomy (Group B), and to compare the oncologic and functional results.

**Methods:**

Clinical prognostic factors, local recurrence rate according to margin status, overall survival and disease-free survival were analyzed.

**Results:**

Margin status is related to recurrence rate in both groups (*p* < 0.05) without significant differences between open and laser cordectomy (*p* > 0.05). The 5 years overall survival and disease-free survival were respectively 90.48% and 85.71% in Group A; 88.14% and 86.44% in Group B (*p* > 0.05). Lower tracheostomy rate, earlier recovery of swallowing function and shorter hospital stay were observed in Group A (*p* < 0.05).

**Conclusions:**

Margin status has a prognostic role in T1a–T1b glottic cancer. Transoral laser surgery showed similar oncologic results of open cordectomy, with better functional outcomes.

## Resumo

**Introdução:**

Cordectomia por laringofissura e cirurgia transoral a laser têm sido propostas para o tratamento do câncer glótico inicial.

**Objetivos:**

O objetivo desse estudo retrospectivo foi avaliar o valor prognóstico do estado da margem em 162 casos consecutivos de carcinoma glótico inicial (Tis-T1) tratado com cirurgia endoscópica a laser de CO_2_ (Grupo A) ou cordectomia por laringofissura (Grupo B) e comparar resultados oncológicos e funcionais.

**Método:**

Foram analisados fatores prognósticos clínicos, taxa de recorrência local de acordo com o estado da margem, sobrevida global e sobrevida livre de doença.

**Resultados:**

O estado de margem está relacionado à taxa de recorrência em ambos os grupos (*p* < 0,05) sem diferenças significativas entre cordectomia aberta e cirurgia a laser (*p* > 0,05). A sobrevida global de cinco anos e a sobrevida livre de doença foram, respectivamente, 90,48% e 85,71% no Grupo A; 88,14% e 86,44% no Grupo B (*p* > 0,05). Menor taxa de traqueostomia, recuperação mais rápida da função de deglutição e menor tempo de internação foram observados no Grupo A (*p* < 0,05).

**Conclusões:**

O estado da margem tem papel prognóstico no câncer glótico T1a-T1b. A cirurgia a laser transoral mostrou resultados oncológicos semelhantes aos da cordectomia aberta, com melhores resultados funcionais.

## Introduction

Laryngeal carcinoma makes up less than two percent of cancers worldwide, even thought the incidence is increasing.^1^, ^2^ Glottic carcinomas represent the majority of laringeal cancer cases.^2^, ^3^ Moreover, “early” glottic cancer (Tis, T1a, T1b, T2) is one of the most curable malignancies in the head and neck. The reason is not only an early diagnosis allowed by the symptom of hoarseness, but also a prevalence less than 1% of patients who develops regional lymph node metastasis, as a result of the glottis peculiar lymphatic drainage.^1^ Different surgical techniques have been described. Cordectomy via thyrotomy is the oldest surgical procedure for the treatment of laryngeal cancer.^4^ At the beginning of the 20th century (1915) Lynch et al. treated glottic carcinoma with endoscopic approach.^5^ In 1972 Strong and Jako introduced CO_2_ laser technology in the surgery of glottic malignancies (transoral laser cordectomy – TLC).^6^ High rates of local control and laryngeal function preservation have been shown for patients with early glottic tumors treated with transoral laser resection or open partial laryngeal surgery. Radiotherapy (RT) is another feasible option for the treatment of glottic cancer.^7^ The evidence suggests that surgery and RT provide higher initial local control rates than exclusive chemotherapy.^8^ Besides cure, laryngeal function preservation has been added as a primary goal of treatment nowadays.^3^, ^4^, ^9^ Additional goals include minimizing the risk of complications and lowering the costs.^3^, ^6^, ^10^

The aim of this retrospective study was to compare the oncologic results (according to the 2010 revised American Joint Committee on Cancer classification)^11^ in a series of 162 cases of early-stage glottic carcinoma (Tis, T1a, T1b) treated with CO_2_ laser endoscopic surgery or laryngofissure cordectomy at our Divisions. Our attention focused on clinical prognostic factors that potentially have a significant impact on local disease control and survival, such as pT classification and margins status. Furthermore, clinical outcomes, such as swallowing function and tracheostomy rate, have been analyzed.

## Methods

Between January 1995 and December 2010, 214 patients with early glottic cancer (Tis, T1a, T1b) were treated at our Divisions. Forty-seven patients underwent exclusive radiotherapy and 167 patients were surgically treated. In our ENT divisions, patients were surgically treated in the majority of the cases. However, the following criteria for indicating surgery or radiotherapy were used: feasibility of cordectomy, sequelae of cordectomy and radiotherapy, patient's comorbidities, and patient's will. Five patients were lost at follow-up (3 underwent laser cordectomy and 2 underwent open cordectomy). One-hundred 62 patients were included in the study. Male/female ratio was 157/5. Mean age was 67.24 ± 10.96 years (age range 41–81 years). Written informed consent was obtained. Exclusion criteria were: presence of nodal and distant metastasis, tumor recurrence (tumor relapse that occurred 6 months or more after previous treatment), previous treatment for laryngeal cancer with laryngeal surgical procedures (except biopsy) or RT (i.e. cordectomies performed for tumor persistence after radiotherapy or surgery, within 6 months after previous treatment).

Patients were treated with two different surgical techniques based on surgeons’ experience. Group A included 86 patients treated with transoral CO_2_ laser-assisted cordectomy. Group B was composed by 76 patients who underwent cordectomy by open approach. The majority of the patients of Group B were treated between 1995 and 2000. Five patients with unsatisfactory glottic exposure (due to ankylosing spondylitis, fracture of cervical spine, mandibular deformity, short thick neck associated with marked prognathism)^12^ underwent open cordectomy after diagnosis with biopsy performed with direct microlaryngoscopy (Group B). Some of the patients who underwent open cordectomy (Group B) were eligible for transoral cordectomy. However, in the first years of this retrospective study, they underwent open cordectomy because of the surgical ability of the surgeon. The two groups were homogeneous for age, sex, tobacco and alcohol consumption, tumor grade and stage, and comorbidities. At diagnosis 117 patients were current smokers, while 37 patients were former smokers; 104 patients were current drinkers. Clinical evaluations and pathological data are summarized in Table 1. Fourteen patients underwent adjuvant radiotherapy. Criteria for choosing adjuvant radiotherapy in positive margin patients were: grading of the tumor, feasibility of a wider excision, sequelae of a wider excision, patient's comorbidities, and patient's will.

**Table 1:** Patients and tumor characteristics.

Characteristics	N° of patients (%)		
	Group A (86 pts)	Group B (76 pts)	Total (162 pts)
*Sex*			
Male	83 (97)	74 (97)	157 (97)
Female	3 (3)	2 (3)	5 (3)
*Smoker*			
Current smokers	57 (66)	60 (79)	117 (72)
Former smokers	24 (28)	13 (17)	37 (23)
Not smoker	5 (6)	3 (4)	8 (5)
*Alcohol consumption*			
Yes	58 (67)	46 (60)	104 (64)
No	28 (33)	30 (40)	58 (36)
*Histological type*			
Squamous cell carcinoma	86 (100)	76 (100)	162 (100)
*Tumor (pTNM VI ed.)*			
Tis	15 (18)	13 (17)	28 (17)
T1a	50 (58)	47 (62)	97 (60)
T1b	21 (24)	16 (21)	37 (23)
*Adjuvant treatment*			
Radiotherapy	8 (9)	6 (8)	14 (9)
*Patients’ mean age*			
Group A	68.54 ± 10.81 years, range 45–81 years		
Group B	65.68 ± 11.45 years, range 41–76 years		
Total	67.24 ± 10.96 years, range 41–81 years		

### Pre-operative staging

Before surgical procedure all patients were examined with fiber optic flexible endoscope. Computed tomography (CT) was performed in all patients with suspect of malignancy. Biopsy for diagnosis was performed when an open cordectomy was expected. In the laser group biopsy was performed in patients with a suspected involvement of anterior commissure, ventricle, arytenoids and/or subglottis. In 7 cases of type I laser cordectomy for probable benign lesion, such as leukoplakia, the pathological diagnosis resulted in Tis or T1a squamous cell carcinoma; therefore a type III or wider laser cordectomy was performed. The clinical staging was conducted according to the American Joint Committee on Cancer classification.^11^ The pathological diagnosis of glottic squamous cell carcinoma was achieved after lesion excision. In case of diagnostic biopsy the ventricle was controlled using 0° and 70° scopes and by palpation under the operative microscope. The feasibility of CO_2_ laser cordectomy was evaluated during endoscopic procedures, whether perfect exposure of the anterior commissure was possible. In all cases the specimens were removed en-bloc. Each specimen was orientated and the margins were identified and marked with ink. The histological grade was determined according to Anneroth's classification.^13^

### CO_2_ laser assisted excision technique

TLC consisted in radical resection of a specimen including the tumor itself and a margin of about 1–2 mm of macroscopically healthy tissue. All surgical procedures were performed under general anesthesia after oro-tracheal intubation with Laser Mackinckrodt Medical tubes with internal diameters ranging from 6.0 to 7.0 mm. Different laryngoscopes were used to obtain laryngeal exposure. A Leika M400E microscope with 400 mm focal lens coupled with a Deka Medical Electronica CO_2_ laser was used. Pulsed energy, mean power in Watt, and excision depth were tailored to carcinoma localization and cordectomy type. Cordectomies were revised according to the European Laryngological Society classification.^14^

Type III cordectomy was performed in 45 patients (52.3%), type IV cordectomy in 20 (23.3%), type V cordectomy in 17 (19.8%) and type VI cordectomy in 4 cases (4.6%). Two patients (2.33%) underwent tracheostomy, to protect lower airways when there was a high risk of post-operative bleeding and/or edema.

### Open cordectomy/cordectomy by laringofissure

External cordectomy was performed through a laryngofissure as described by Buck^15^: vertical cervical incision in the middle line, section of the white line to expose the larynx and trachea, opening of the thyroid prominence and excision of the diseased (neoplastic) vocal cord together with its paraglottic space. During surgical procedure, 6 patients (7.89%) underwent tracheostomy, using Ciaglia's technique or Portex Griggs’ tracheostomy kit, to protect lower airways when there was a high risk of post-operative bleeding and/or edema.

### Margins status

Intraoperative biopsies were performed only in case of suspicion of incomplete tumor resection. Histological analysis of resection margins was performed by the same team in all cases, with the same technique and criteria. Surgical specimens were fixed in 4% formaldehyde for 48 h, inked on their superficial (mucosal) and deep sides with two different colored inks before inclusion in their entirety. Then they were sliced axially (parallel to the vocal folds) with 3–4 mm thickness. Positive margins were defined by “in situ” or invasive carcinoma in contact with the margin, close margins was characterized by 1 mm or less between margin and tumor, and negative margins was characterized by a distance greater than 1 mm. All specimens were reassessed by a pathologist.

### Follow-up

In patients with negative margins, clinical evaluations (including flexible laryngoscopy, videostrobolaryngoscopy, or both) were performed every 3 months in the first year, every 4–6 months during the second year, and annually for the next years. Patients with close margins, positive margins or a precancerous lesion (mild to moderate laryngeal intraepithelial neoplasia) were assessed every month for first 6 months, every 2 months for the next 6 months, every 3 months for the next year, every 6 months in the third year and annually for the next years. Repeated microlaryngoscopy and excisional biopsies were performed only when relapses were suspected. The mean follow-up period was 76.6 months (range 25–148 months). All patients had at least a 24 month follow-up period. One hundred-twenty-two patients had at least 5 year follow-up period: 63 of Group A (10 Tis, 37 T1a, 16 T1b) and 59 of Group B (8 Tis, 39 T1a, 12 T1b).

### Statistical analysis

Graphpad Prism for Windows, version 5, was used for statistical analysis. The Kaplan–Meier method and the Cox regression test were used for survival analysis curves. Comparison among qualitative variables was performed by means of *χ*^2^-test (or Fisher's exact test when necessary). All statistical tests received the same level of significance of 0.05.

## Results

Concerning patients and tumor characteristics (Table 1), no statistically significant difference was observed between the two groups (*p* < 0.05).

Positive specimen margins were found in 11 patients in Group A (one patient underwent salvage surgery, 8 patients were treated with radiotherapy and 2 had a watchfull-waiting follow-up), and 8 patients in Group B (6 patients underwent adjuvant radiotherapy and 2 had a watchful-waiting follow-up). Patients with definitive positive margins had a microscopic invasion of the superficial and/or deep margin, so the surgeon did not suspect it at operative time. Positive intraoperative margins were found in 3 patient of Group A, who underwent a wider laser surgery, and 2 patients in Group B, who underwent a wider surgical excision.

Definitive histological exam was negative for carcinoma in 2 patients of Group A (2.32%) and 3 patients of Group B (3.94%) (*p* = 0.10). In these cases the whole tumor was resected during the biopsy procedure.

In Group A, recurrence of disease occurred in 2 patients out of 86 (2.32%) within 2 years of follow-up, while in 6 cases (6.97%) recurrence was observed within 5 years of follow-up. Concerning Group B, 1 patients out of 76 (1.31%) had recurrence within 2 years, and 5 patients out of 76 (6.58%) within 5 years. The difference was not statistically significant (*p* = 0.10). Margin status is related to recurrence rate in both groups and it is reported in Table 2. No statistically significant difference was found between groups according to margin status. In Group A, 5 patients affected by recurrence underwent salvage surgery with laser technique (2 patients), supracricoid laryngectomy (1 patient) or total laryngectomy (2 patients), while 4 patients were treated with chemoradiotherapy, according to patients’ comorbidities and will. Concerning Group B, salvage surgery with partial or total laryngectomy was used in 4 patients with recurrence (2 patients underwent supracricoid surgery and 2 patients total laryngectomy). In Group B, chemoradiotherapy was performed in 2 cases and in one case recurrence was treated with radiotherapy alone.

**Table 2:** Local recurrence rate according to margin status.

Margin status	Group A		Group B		*p*
	N° pts	Local recurrence rate	N° pts	Local recurrence rate	
Negative	60	5 (8.33%)	64	5 (7.81%)	0.80
Close	12	1 (8.33%)	2	0 (0.0%)	0.07
Positive	14	3 (21.42%)	10	2 (20.00%)	0.21
Entire sample	86	9 (10.20%)	76	7 (9.21%)	0.10

The 2-year overall survival (OS) rate was 97.67% in Group A and 96.05% in Group B. Comprehensively, 5 out of 162 patients (2 in Group A and 3 in Group B) died within 2 years, for cardiovascular accident or second primary tumor (lung). No exitus was related to glottic cancer. The 5 year overall survival rate was 90.48% in Group A and 88.14% in Group B. Log-rank test shows that this difference was not statistically significant (*p* = 0.30). Only one patient out of 162 died for related tumor reason: bleeding occurred during salvage surgery for laryngeal tumor recurrence. Other deaths were related to cardiovascular accidents or lung and esophageal malignancies.

The disease free survival (DFS) rate at 2 years was 96.51% in Group A and 96.05% in Group B. The disease free survival at 5 years was 85.71% in Group A and 86.44% in Group B. Log-rank test shows that this difference was not statistically significant (*p* = 0.25).

**Figure 1:**
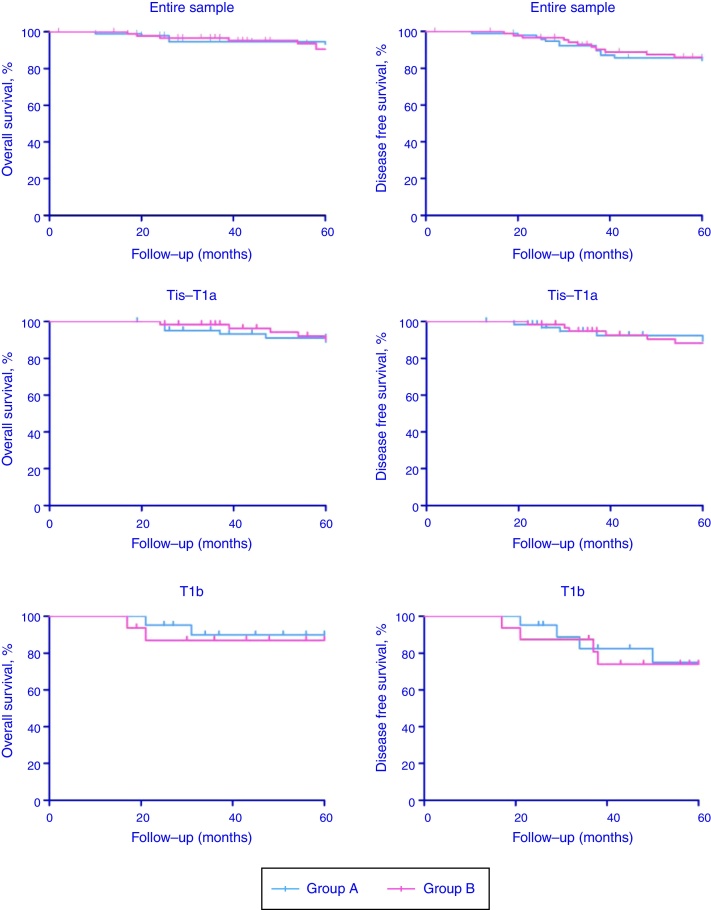
Overall survival (OS) and disease free survival (DFS) Kaplan–Meyer curves according to CT stage.

Patients of Group A and Group B were stratified in two subgroups according to cTNM classification (Tis-T1a and T1b). Comprehensive oncologic results (overall survival rate, OS; disease-specific survival rate, DFS) stratified according to CT stage have been summarized in Table 3 and Fig. 1 (*p*-values for Tis-T1a and T1b were the followings: 0.58 and 0.53 for OS and 0.22 and 0.74 for DFS, respectively). Organ preservation was similar in the two groups (2 total laryngectomy were performed in each group for recurrent disease).

**Table 3:** Overall survival (OS) and disease free survival (DFS) according to CT stage.

Follow-up	Group A				Group B			
	OS		DFS		OS		DFS	
	2 years	5 years	2 years	5 years	2 years	5 years	2 years	5 years
Entire sample	84 (97.67%)	57 (90.48%)	83 (96.51%)	54 (85.71%)	73 (96.05%)	52 (88.14%)	73 (96.05%)	51 (86.44%)
Tis-T1a	64 (98.46%)	43 (91.49%)	63 (96.92%)	42 (89.36%)	59 (98.33%)	42 (89.36%)	59 (98.33%)	42 (89.36%)
T1b	20 (95.24%)	14 (87.50%)	20 (95.24%)	12 (75.00%)	14 (87.50%)	10 (83.33%)	14 (87.50%)	9 (75.00%)

OS, overall survival; DFS, disease free survival.

Functional outcomes, such as mean time needed to restore swallowing function and tracheostomy rate, were evaluated in both groups and compared. In patients of Group A, mean time of swallowing function recovery was 1.76 days (range 1–4 days); while in Group B it was 5.51 days (range 3–7 days). Tracheostomy was performed in 2 patients (2.33%) of group A and in 6 patients (7.89%) of Group B. No pharyngeal fistulae was observed. Both these differences were statistically significant (*p* < 0.05). Hospital stay was significantly reduced in patient of Group A (mean time: 3.19 days) versus patient of Group B (6.34 days) (*p* < 0.05). These results are reported in Table 4.

**Table 4:** Functional outcomes according to treatment.

Functional outcomes	Group A	Group B	*p*
Tracheostomy (%)	2.33	7.89	0.04
Swallowing function recovery (days)	1.76 ± 1.23	5.51 ± 2.04	0.02
Hospital stay (days)	3.19 ± 1.85	6.34 ± 2.12	0.04

## Discussion

The role of open surgery for the management of laryngeal cancer has been greatly diminished during the past decade. The development of transoral endoscopic laser microsurgery (TLC), the improvements in delivery of radiation therapy (RT) and the advent of multimodality protocols, have supplanted the previously standard techniques of open partial laryngectomy for early cancer.^16^

Anatomically, early laryngeal cancer is defined as an invasive cancer confined to the three layers of the lamina propria, and not invading the adjacent muscles and cartilages.^17^ However, in the literature, the term is generally used for Tis, T1, T2 lesions as a group.

According to literature,^18^, ^19^, ^20^, ^21^, ^22^ our study showed that the oncologic results of laser surgery for selected patients in the treatment of Tis–T1 laryngeal cancer are equivalent to those achieved with open partial laryngectomy with less morbidity and usually without the need for tracheostomy. The current literature is now concentrating on the comparison of laser surgery and radiotherapy.

Our study focused on margin status and a prognostic role was proven in both group of patients. Concerning management of patients with positive or close margins, nowadays there is no consensus about post-operative strategies. Some authors recommended biopsy;^23^ it is not unusual that final histological analysis is less favorable than the extemporaneous analysis, discovering non-negative margins. The problem for the clinician is then to decide between surveillance, surgical revision and radiation therapy.^24^ Some studies found that positive margins after careful resection in macroscopically healthy tissue are not a pejorative factor for overall or recurrence-free survival in T1a patients endoscopically treated.^25^, ^26^, ^27^ Therefore, adjuvant treatments, such as radiation therapy or surgical revision, do not seem indicated. In case of macroscopically negative, but microscopically positive margins, some authors recommend endoscopic control with targeted biopsy under general anesthesia 10 weeks after surgery.^28^, ^29^, ^30^

Other authors observed that positive margins after tumor resection are associated with a higher rate of local recurrences.^31^, ^32^, ^33^ Ansarin et al. found that when the margins were positive, the incidence of local recurrence was higher and DFS was lower (76.7% at 84 months) compared to patients with free margins. These findings indicate that additional treatment should always be given if positive margins are found.^34^

In our study positive margins were found in 24 patients; 17 of them underwent adjuvant RT while 5 were treated with surgery. Two patients were managed with watchful waiting approach because of anesthesiological problems and radiation therapy refusal. According to literature, local recurrence rate was higher in patients with positive margins.^35^ We did not find statistical differences in local recurrence rate between laser and open surgery. In 2 patients of Group A and 3 patients of Group B definitive histological exam was negative for carcinoma.

Beyond oncologic results, other evaluated outcomes in literature are morbidity, vocal function, hospitalization length and costs. When performing cordectomy by laryngofissure, the thyroid cartilage and endolaryngeal soft tissues are divided. Sometimes after surgery there could be a compromise of the airways and therefore a need for temporary tracheotomy. With endoscopic resection, tracheostomy is very rarely indicated. Avoiding tracheotomy and preserving the prelaryngeal muscles can facilitate a quick, safe recovery of swallowing.^36^ Functional results with TLC are generally better than those of conventional open surgery, in terms of time needed to restore swallowing, tracheotomy rates, incidence of pharyngeal fistulae and shorter hospital stays.^37^, ^38^ These functional benefits may be attributed to the more conservative nature of the endoscopic technique, since normal tissues are not interrupted during the procedure.^36^ In fact, in transoral laser cordectomies, the functional sequelae are exclusively voice-related. Difficulties in swallowing liquids after the procedure are temporary and resolve spontaneously in a few days.^39^ Our results confirmed the data reported in literature regarding need for tracheostomy and swallowing function recovery.

In literature and in our study, the use of CO_2_ laser surgery was associated with a shorter hospital stay and earlier return to work than laryngofissure cordectomy.^40^ For these reasons, CO_2_ laser cordectomy resulted as a cost-effective treatment modality if compared to open cordectomy or radiotherapy.^41^, ^42^, ^43^ In particular, Cragle and Mandeburg observed that CO_2_ laser cordectomy was almost 58% cheaper than radiotherapy with the same oncologic results. In 1994, a study of Myers obtained a similar result: CO_2_ surgery is 70% cheaper than radiotherapy.

The costs included hospital admission and stay, materials and surgical time, as well as healthcare and non-healthcare personnel associated with the procedure. Specifically, it indicated that transoral laser cordectomy was less expensive than laryngofissure cordectomy. Furthermore, open cordectomy costs increase because of the later return to work.

CO_2_ laser cordectomy and open cordectomy afford optimal oncologic radicality for early glottic cancer. Besides cure, compared to laryngofissure, CO_2_ laser cordectomy offers different advantages. The absence of need for feeding tube or tracheotomy after CO_2_ laser procedure eliminates two of the great stigmas regarding laryngeal cancer treatment. Furthermore, a more conservative approach guarantees a shorter hospitalization and lower costs. Finally transoral approach is related to a lower risk of complications.

## Conclusions

Margin status has an important prognostic role both in open cordectomy and in CO_2_ laser cordectomy. Therefore additional treatment should be considered in case of positive margins; in order to reduce recurrence rate and consequent need of more aggressive surgery. Concerning management of patients with close margins, further studies are necessary to obtain a consensus about post-operative strategies.

## Conflicts of interest

The authors declare no conflicts of interest.
